# Trans-iliac-sacral-iliac-bar procedure to treat insufficiency fractures of the sacrum

**DOI:** 10.4103/0019-5413.53454

**Published:** 2009

**Authors:** P Vanderschot, M Kuppers, A Sermon, L Lateur

**Affiliations:** Department of Traumatology, University Hospital Gasthuisberg, Herestraat 49, B-3000 Leuven, Belgium; 1 Department of Radiology, University Hospital Gasthuisberg, Herestraat 49, B-3000 Leuven, Belgium

**Keywords:** Elderly, osteoporosis, sacrum, fracture, insufficiency, minimally invasive, bar

## Abstract

**Background:**

Osteoporosis is an increasing problem attributed to the greater longevity of the population and the incidence of fractures related to osteoporosis. The presence of osteoporotic bone, comorbidities, and functional status of the patient require adequate solutions to improve the clinical outcome of sacral insufficiency fractures. Conservative treatment by means of prolonged bed rest and analgesics are associated with increased risks and complications. A sacroplasty significantly improves the functional outcome. We describe the trans-iliac-sacral-iliac-bar (TISIB) procedure and our clinical experience to treat insufficiency fractures of the sacrum.

**Materials and Methods:**

The records of 19 consecutive patients with a mean age of 71.7 years (range: 57-82 years) who had been managed with a TISIB procedure from 2005 till 2007 were reviewed retrospectively. There were 15 females and 4 males. Predisposing factors for sacral insufficiency fractures were osteoporosis (n = 12, 63%), radiotherapy (n = 6, 32%), and rheumatoid arthritis (n =1). Diagnosis with a mean delay of 3.7 months was mainly made by CT. All patients were preoperatively and at follow-up assessed by means of the visual analogue score (VAS), analgesic consumption, and the ability to perform activities of daily living (ADLs) using a 5-point pain scale: 1, without pain; 2, mild pain; 3, moderate pain; 4, severe pain and, 5 unable to perform ADLs because of pain.

**Results:**

The average duration of postoperative follow-up was 9 months (range: 3–24.5 months). No neurological complications occurred during the surgery. A postoperative radiographic study showed a well-positioned bar in every case. The mean VAS improved 44.7 mm (preoperative: 67.8; at follow-up: 23.2). Fifteen patients (79%) consumed narcotic analgesics before surgery, and only one (5%) at follow-up; two patients (10%) consumed NSAIDS before surgery and three (15%) after. Two patients (10%) consumed minor analgesics before, and 11 (58%) after the procedure. Finally, four patients (21%) were not taking any analgesics at follow-up. Before surgery, 9 patients (47%) were able to perform ADLs with a pain score of 4; 6 (32%) with a score of 3, and 4 (21%) a score of 2. At follow-up 1 (5%) did have a score of 4; 1 (5%) a score of 3, 8 (42%) a score of 2 and 9 (47%) a pain score of 1.

**Conclusion:**

A TISIB procedure relies on the principles of fracture treatment: fracture stabilisation and compression. The incapacitating problem of an insufficiency fracture of the sacrum can be elegantly solved by means of this minimally invasive procedure. A near-immediate improvement is noticed when looking at the VAS score, analgesics consumption, and the ability to perform ADLs.

## INTRODUCTION

Increasing knowledge of fragile bone has been gained by noninvasive mineral assessments. Its future importance seems to be twofold. First, it seems likely that patients with low bone minerals will be treated to try to increase their bone minerals or at least to keep it steady. Second, in the case of osteoporosis, adapted surgical procedures must be taken into consideration, which enable elderly people to return to their normal daily life within an acceptable time frame. It is obvious that an osteosynthesis with plate and screws which have their definite indications in younger patients may be replaced by salvage procedures, such as a vertebroplasty, sacroplasty, PMMA cement augmentation of screws,^3^ and cable fixation of acetabular fractures with total hip arthroplasty.^4^

The current standard of care for people with sacral insufficiency fractures includes the administration of analgesics and, in some patients, prolonged bed rest.^1^,^5^–^7^ Prolonged immobilization of elderly patients is known to be associated with significant risks and complications. Sacroplasty, a variant of vertebroplasty, is a relatively new minimally invasive percutaneous procedure that has been described as an alternative to the conventional therapy.^1^,^8^,^9^

In this article, we present a trans-iliac-sacral-iliac-bar (TISIB) procedure to treat insufficiency fractures of the sacrum by means of a minimally invasive procedure and discuss the clinical outcome of 19 patients.

## MATERIALS AND METHODS

### Clinical data

Between June 2005 and January 2008, 19 patients were treated for an insufficiency fracture of the sacrum by means of a TISIB at the Catholic University of Leuven, Belgium.

Of a total of 19 patients, there were 15 women with a mean age of 71.5 years (range: 57–82) and only four men with a mean age of 68 years (range: 59–75). Osteoporosis was seen in 12 cases, of which all but one were female. One male had a medical history of rheumatoid arthritis, and six patients had a history of radiotherapy for different kinds of malignancies: rectum (2), cervix (2), endometrium (1) and multiple myeloma (1). Corticosteroid intake was noted in two cases in the group of osteoporosis and in one with rheumatoid arthritis.

A minor trauma could be noticed in four patients (20%). In all the others, the pathology was attributed to an occult onset. Clinical expression was low back pain in five patients and truncated sciatica or sciatica of S1 topography in fourteen patients. The Visual Analogue Scale (VAS) score,^10^ analgesic consumption, and the ability to perform activities of daily living (ADLs)^11^ was evaluated preoperatively and at follow-up after a mean of 9 months (range: 3–24) [Table 1].

**Table 1:** Clinical data of every patient: Number, sex, age, predisposing factors (osteoporosis, radiotherapy, rheumatoid arthritis, and corticosteroids) and clinical appearance (low back pain, truncated sciatica, sciatica S1 topography).

				Insufficiency fracture			Preoperative status			Follow up			
						
Sex	Age	Etiology	Symptoms	Loc.	XR	Diagnosis	VAS	Analgesics	ADL	Mths	VAS	Analgesics	ADL
F	70	osteoporosis	TS/ S1	B	-	CT	72	Narcotic	2	4.8	23	Minor	1
F	79	osteoporosis	LBP	B	-	CT	53	Minor	4	11.5	44	Minor	2
F	63	osteo/corticosteroids	TS/ S1	B	-	Scint./CT	71	Narcotic	4	15	24	None	2
F	77	osteoporosis	TS/ S1	B	-	CT	74	NSAIDS	3	5.1	22	None	1
F	64	osteoporosis	LBP	B	-	CT	59	Narcotic	2	3.6	34	Minor	2
F	83	osteoporosis	LBP	B	+	CT	58	Narcotic	2	11	24	Minor	2
M	75	osteoporosis	LBP	B	-	CT	56	Narcotic	4	4	22	Minor	1
F	76	osteoporosis	TS/ S1	B	-	Scint./CT	66	Narcotic	3	7	35	Minor	2
F	71	osteoporosis	TS/ S1	B	-	CT/MRI	73	Narcotic	3	4	10	None	1
F	75	osteoporosis	TS/ S1	B	-	CT	78	Narcotic	4	3	10	None	1
F	65	osteoporosis	TS/ S1	B	-	CT	76	Narcotic	4	3	12	Minor	1
F	57	osteo/corticosteroids	TS/ S1	B	-	Scint./CT	82	Narcotic	4	18.3	25	NSAIDS	2
M	76	RA/corticosteroids	TS/ S1	B	+	CT	83	Narcotic	4	4	21	NSAIDS	1
F	66	RT	TS/ S1	B	-	Scint./CT/MRI	65	Minor	2	23	17	Minor	1
F	83	RT	TS/ S1	B	+	CT	72	Narcotic	4	18.3	32	NSAIDS	4
M	59	RT	TS/ S1	U	-	CT/MRI	67	NSAIDS	3	3.6	24	Minor	1
M	62	RT	TS/ S1	B	+	CT	64	Narcotic	3	24.5	23	Minor	2
F	76	RT	LBP	B	-	CT/MRI	45	Narcotic	3	4.3	24	Narcotic	3
F	68	RT	TS/ S1	B	-	Scint./CT	76	Narcotic	4	6	15	Minor	2

Data related to the fracture: location: uni- (U) or bilateral (B); diagnostic procedure: standard radiographs (XR), CT scan, MRI and bone scintigraphy. Preoperative status: visual analogue score (VAS), analgesics consumption (narcotic, non steroidal anti-inflammatory drugs, and minor analgesics) and ability to perform activities of daily living (ADLS) using a 5-point scale. At follow up, the time of follow up (months), VAS, analgesics consumption and, ability to perform ADLs.

The VAS, presented as a 10-cm line, was used to indicate pain intensity. The mean visual analogue score preoperatively was 67.8 mm (range: 45–83). Narcotic analgesics (fentanyl: Durogesic®; or buprenorfine: Transtec®) were administered in 15 patients, nonsteroidal anti-inflammatory drugs (NSAIDs) and minor analgesics (paracetamol: Dafalgan®, Panadol®, or Perdolan®) in two patients.

The ability to perform ADLs was evaluated with a 5-point scale: 1, able to perform ADLs without pain; 2, able to perform ADLs with mild pain; 3, able to perform ADLs with moderate pain; 4, able to perform ADLs with severe pain; and 5, unable to perform ADLs because of pain.^11^ Nine patients (47%) were able to perform ADLs with severe pain (score: 4). All of them were almost bedridden and care-dependent. Six patients (32%) were moderately impaired (score: 3), and four (21%) experienced mild pain during their ADLs (score: 2).

A plain radiograph of the pelvis showed a fracture without sclerosis of the sacrum in only four cases (20%). A CT scan was done in all patients. In only one case, a CT scan was unhelpful. In that case, an additional MRI was necessary to demonstrate a bilateral lesion of the sacrum. In three patients, an MRI was used to exclude preexisting metastatic lesions of the pelvis. Eighteen patients had a bilateral and one patient had a unilateral insufficiency fracture of the sacrum.

Time from symptoms to diagnosis averaged 3.7 months (range: 0.5-9). However, in presence of a minor trauma (four patients) the diagnosis was made after a mean of 3 weeks (range: 2-5). In presence of an occult onset (15 patients, 79%), the mean delay of diagnosis was 3.7 months (range: 1-9).

Only one patient was treated after a delay of diagnosis of three months, and the others were scheduled for surgery as soon as possible. All surgical procedures were done using fluoroscopic guidance. The position of the bar inside the bone was checked in each patient: 12 times by means of a standard radiography of the pelvis including inlet and outlet views and seven times by means of a CT scan.

### Preoperative planning, operative procedure, and limitations

A preoperative planning of the iliac-sacral-iliac path is mandatory to understand the unique location of the path in a given patient. Indeed, the localization of the safe area at the level of S1 varies from one patient to another. Furthermore, there is no common safe area with a diameter of at least 5 mm to rely on for both males and females.^12^

A preoperative planning was done using a navigation system or Picture Archiving and Communication System (PACS) software.

**Figure 1:**
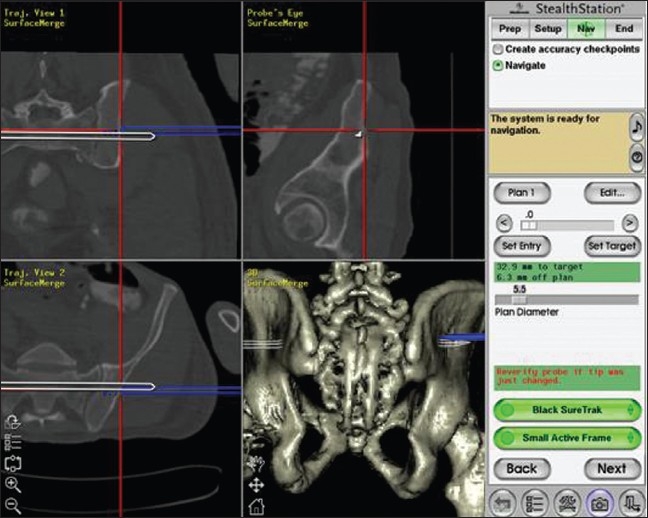
StealthStation™, Treatment Guidance Platform. Tri-axial reformats (coronal, sagittal and axial anatomical planes) and a three-dimensional reconstruction are displayed. The optimal Iliac-sacral-iliac path (white bar) is indicated, diameter 5.5mm. According to the preoperative plan, a path can be drilled using the Sure Track (blue)

When a navigation system is preferred, a CT scan of the pelvis with continuous, nonoverlapping, 2 mm thick axial cuts is performed as per the protocol of the navigation system (e.g., StealthStation™, Treatment Guidance Platform (Sofamor Danek, Memphis, TN). The image data are archived to an optical disk and imported into the software of the StealthStation™. Three-dimensional reconstruction of the pelvis was performed. The software displays tri-axial reformats simultaneously: images in sagittal, coronal, and axial anatomical planes. The entry and target of the trajectory of iliac-sacral-iliac path are determined. It starts at the narrowest area, the level of the neuroforamina, and extends to both iliac wings in the same axis. The length and largest diameter of the path is measured. After clarifying the location of the trans-iliac-sacral-iliac path, length, and diameter at the desired level, the system accurately guides the surgeon into the right direction during the surgical procedure [Figure 1].

**Figure 2:**
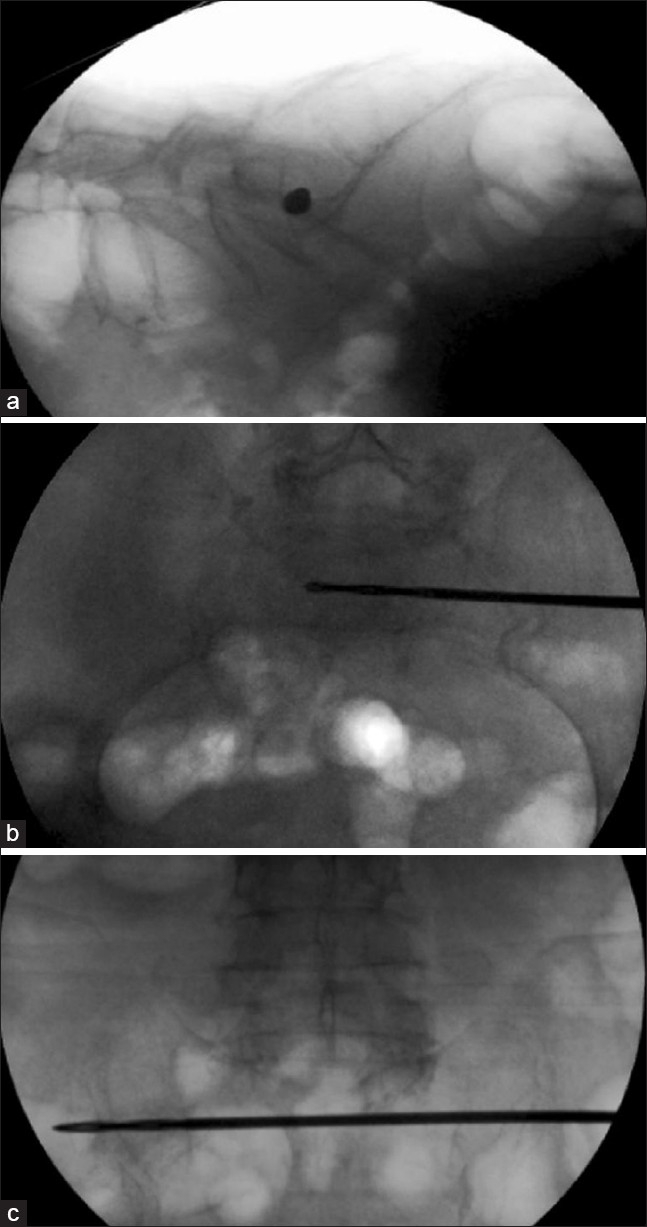
TISIB procedure with fluoroscopic guidance. In prone position, a lateral view is obtained to mark the entry point at the level of the skin and bone (a) An iliac-sacral-iliac path is progressively drilled under control of inlet (b) and outlet views (c) of the pelvis

**Figure 3:**
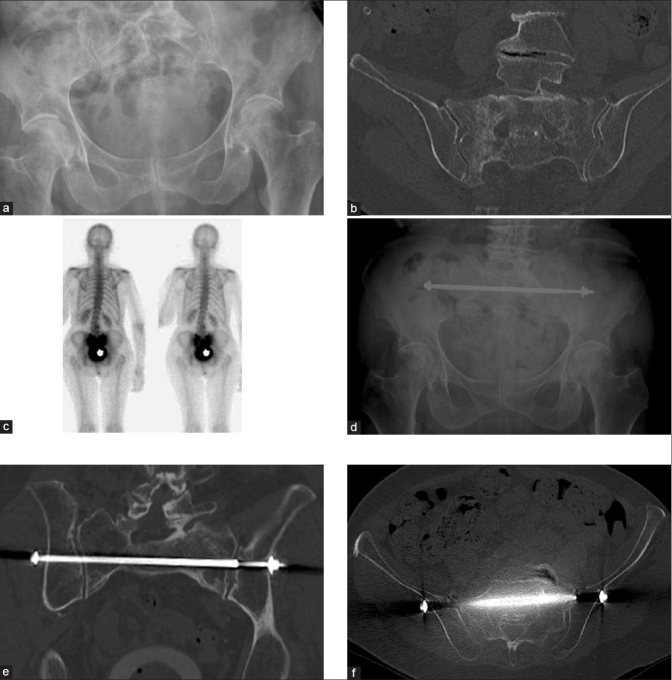
76 years old women with complaint of incapacitating low back pain radiating to the left lower extremity (S1 topography). Plain radiographs were unhelpful to make a diagnosis (a). An axial CT scan with coronal and sagittal reconstructions showed bilateral insufficiency fractures of the sacrum (b). A bone scan using 99mTc-MDP tracer showed an increased uptake at the level of both sacro-iliac joints (c). A TISIB-procedure has been performed since the insufficiency fractures occurred bilaterally (d). A postoperative CT scan with axial reconstructions showed a well-positioned bar at the level of S1 (e). A postoperative CT scan with coronal reconstructions showed a well-positioned bar at the level of S1 (f).

When PACS is preferred, the characteristics of the iliac-sacral-iliac path are determined using tri-axial reformats of a CT scan. As the data of a given patient cannot be transferred to the patient during surgery, fluoroscopic guidance is mandatory to perform the surgery. The skin incision and the entry point of the drill (3.2 mm) inside the “safe area” of S1 are defined with the C-arm in the lateral position as mentioned by M. Tile.^12^ The anteroposterior (AP), inlet, and outlet views of the pelvis continuously allow the surgeon to control the direction of the drill axis while creating an iliac-sacral-iliac path [Figure 2a–c]. After inserting a K-wire in the path, a 5-mm cannulated drill is used to enlarge the trajectory to the appropriate diameter. A bar (Ulrich GmbH and Co. KG, Ulm, Germany) with an appropriate length (range: 170–210 mm, increasing by 10 mm) and a diameter of 5 mm is inserted and tightened for final stabilization and compression [Figure 3a–f].

Finally, it is important to realize that a TISIB-procedure is not always possible. This can be related to the complexity and individual variability of the sacrum itself but also to factors related to the patient. In a previous article,^13^ the iliac-sacro-iliac path (5 mm or more) has been studied in 20 patients of which 10 were men and 10 were women. It was clearly demonstrated that a TISIB procedure would have been possible to perform at the level of S1 and S2 in all men, but only in five women at the level of S1 and in eight women at the level of S2. On the other hand, when using fluoroscopic guidance, anatomical landmarks should be clearly visible on the lateral, inlet, and outlet view. This is not always possible, especially in presence of extreme osteoporosis or morbid obesity. In such cases a conservative management, sacroplasty or a uni- or bilateral screw fixation at the level of the sacro-iliac joint using a navigation system can be considered.

## RESULTS

The mean time of the operative procedure was 60 min (range: 40–180). No intraoperative iatrogenic neurological complications occurred. Two haematomas attributed to the laceration of a branch of the superior gluteal artery needed to be drained.

In each patient, a well-positioned implant inside the bone could be observed. In the early postoperative period, the position was checked by means of lateral, inlet, and outlet views of the pelvis. In 7 patients, a CT was performed at follow-up of at least three months after the procedure, which showed a well-positioned implant with signs of fracture healing.

The near-immediate relief of pain in the postoperative period spontaneously allowed the patients to mobilize full weight bearing. All of them were already mobilizing during their admission in the surgical ward. After a mean hospital stay of five days (range: 3-10), 16 were discharged home or a nursery home. Only three were referred to another department to continue their medical treatment of comorbidities.

The mean VAS score[10] was 67.8 mm (range: 45-83) preoperatively and 23.2 (range: 10-44) at a mean follow up of 9 months (range: 3–24.5 months). A mean improvement of 44.6 could be noticed.

Four patients did not need any analgesics in the group of osteoporosis, seven needed minor analgesics, and only one needed NSAIDs on a regular basis. Of the remaining seven patients, two needed NSAIDS on a regular basis, i.e one patient with rheumatoid arthritis and one treated for a carcinoma. The only one patient (case: 18), who died after 4.3 months of follow-up, was still on narcotic drugs due to comorbidities. The other four only needed minor analgesics.

One patient with a score of 4 (case 15), and one with a score of 3 (case 18) did have a severe and moderate impairment of ADLs respectively. Both were treated for a carcinoma and required age related assistance by a caregiver. Eight patients had a score of 2 and were able to perform ADLs with mild pain. The remaining 9 patients did not experience pain during their ADLs.

In the group of osteoporosis, five patients were able to walk unaided. Of these, seven were using a simple walking aid (stick or frame) with one of the patients only needing a walking-aid when outdoors. Use of a walking aid was related to spinal disorder in three, hip arthroplasty in one and age related in two patients (83 and 79 years old). None required walking assistance by a caregiver. In the other group of seven patients (rheumatoid arthritis and radiotherapy for carcinoma), two required walking assistance by a caregiver (case 15 and 18), two were able to walk with a stick or frame and three were walking unaided.

## DISCUSSION

Sacral insufficiency fractures are relatively common injuries that are associated with severe and functionally debilitating pain.^5^–^7^,^14^ Most *insufficiency fractures of the sacrum* occur in women (94.3%) of advanced age (mean age: 70.6 years).^15^ The most common risk factor is osteoporosis. Patients may have undergone prolonged corticosteroid treatment.^16^ Other risk factors mentioned by Finiels *et al.*^15^ include radiotherapy,^17^ osteomalacia, rheumatoid arthritis,^18^ hip arthroplasty,^19^ and fluoride treatment.

Sacral insufficiency fractures occur either spontaneously or after a trivial traumatic episode. In about 30%, trauma is cited as a causative factor.^15^ Vertical compression forces appear to be the cause of insufficiency fractures in the osteoporotic sacrum.^20^

Clinical expression is low back pain and groin pain in presence of associated pelvic ring fractures anteriorly.^21^ Truncated sciatica and sciatica of S1 topography may be observed. Furthermore, neurological complications such as motor deficit of the lower limbs and urinary incontinence have been rarely reported.^22^,^23^

Diagnosis of sacral insufficiency fractures is difficult since the onset is mild, and usually the discomfort is attributed to degeneration of the lumbar spine. This may delay the diagnosis by 1 month^24^ and 2.07 months^15^ as reported in the literature.Plain radiographs are usually unhelpful or may be misleading. A sensitivity of 37%^24^ and 34.3%^15^ has been reported. Scintigraphy has a very high sensitivity of 92.6%,^24^ 97.3%,^15^ and 100%.^25^ A complete or partial H-shaped pattern, described by Ries^26^ is the most common pattern and the combination of concomitant sacral and parasymphyseal uptake is considered as characteristic for insufficiency fractures.^27^ CT scan shows a fracture line often surrounded by prominent sclerosis.^28^ A diagnostic sensitivity of 95.9%^15^ and at variance a vacuum phenomenon at the level of the insufficiency fractures of the sacrum and/or in the sacroiliac joints has been reported.^27^ MRI sensitivity is estimated at 97.2%^15^ but not specifically in detecting sacral insufficiency fractures.^29^ It is especially useful for excluding pre-existing metastatic lesions.^30^

In many locations, osteoporotic fractures may need special solutions. It is obvious that osteosynthesis with plates and screws which have their definite indications in younger patients may be replaced by adapted surgical procedures.

Sacro-iliac screws are subjected to axial as well as bending loads. The holding power of a screw in bone is associated with the mechanical properties of the screw itself (thread depth, pitch and root diameter^31^) and the shear strength of the bone (volume of bone the threads capture).^32^ The length of the screw has a dramatic effect on the pull-out strength.^33^ Nevertheless, loosening or pull-out of sacroiliac screws in osteoporotic bone is a well known phenomenon even in young patients.^34^,^35^ Polymethylmethacrylate-augmented pedicle screw fixation results in a significant increase in the axial pull-out strength of augmented pedicle screws in both primary and revision procedures.^3^

The biomechanical benefits of TISIB are twofold. The stability of the procedure does not rely on the holding power of screws. By going through the vertebral body of S1, the two fracture sides are simultaneously stabilized. Furthermore, maximal stabilization is achieved by going through the entire length of the still intact bone at the level of the vertebral body of S1. Finally, the compression exerted at the level of the fracture sites depends entirely on the implant instead of the holding power of the screw thread.

The treatment of insufficiency fracture of the sacrum is changing over time. Besides the early conservative treatment, other minimally invasive procedures were introduced over the last years.

The standard of care for people with sacral insufficiency fractures includes the administration of analgesics of varying efficacy and, in some patients, prolonged bed rest.^1^,^5^–^7^,^36^ It has been reported that sacral insufficiency fractures may require up to 12 months to heal.^1^,^6^ Prolonged immobilization of elderly patients is known to be associated with significant risks and complications, including pneumonia, urinary tract infections, and pressure sores, as well as deep venous thrombosis and associated pulmonary embolus.^36^ Outcomes of conservative management have been mixed in several reported case series, with some studies reporting recovery in all patients, and others reporting a subset of patients with longer-term disability.^7^,^14^,^21^,^25^,^37^ Indeed, one case series describes poor long- term prognoses in most patients who were followed clinically after diagnosis and conservative treatment of sacral insufficiency fractures.^38^ Other case series also report costly prolonged hospitalization, with a mean admission of 21 days^25^ and 60 days.^7^ Functional outcomes after conservative management seem to vary among case reports. Although some studies report improvements in symptoms including mobility status, within 3-5 weeks, others report recovery times of several months, with one case series reporting a return to independent mobility after an average of 11 months.^21^,^39^,^40^

Sacroplasty, a variant of vertebroplasty, is a relatively new minimally invasive percutaneous procedure that has been described as an alternative to conventional therapy.^1^,^9^,^41^ Many patients with sacral insufficiency fractures report decreased pain and increased mobility within hours after sacroplasty.^1^,^9^,^41^

The periods of reductions in pain and increases in the ability to ambulate and perform various ADLs after the procedure are confirmed in the literature. In one case series, the author reports symptomatic improvement in patients followed for up to 14 weeks after sacroplasty.^41^ A second case series reports symptomatic improvement in patients followed for up to 9 months.^1^ In the series of Whitlow *et al.*^2^ patients reported decreased pain and increased ability to ambulate and perform ADLs for a mean duration of more than 1.5 years (range, 254-1268 days; median, 647 days) after undergoing sacroplasty.

Deen *et al.*^42^ reported favourable clinical outcomes in three patients using a balloon kyphoplasty at the level of the insufficiency fracture of the sacrum. The VAS pain score improved by four points in each case. The functional status improved and analgesic requirements decreased in all three patients. There were no complications associated with the procedure.

In present report, it was difficult determine the delay of diagnosis when no minor trauma had been reported. Many of these patients did report symptoms for six months or more and their analgesic consumption progressively increased over time. Many of these patients were referred to our department after failure of conservative management.

Nineteen patients were treated by means of a TISIB-procedure. All patient experienced near-immediate relief of pain and improvement in mobility after the procedure. This relief of pain spontaneously allowed the patients to mobilise full weight bearing in the postoperative period. The functional ability of all patients gradually improved over time with a simultaneous decrease of analgesics consumption.

## CONCLUSION

Insufficiency fractures of the sacrum are not that uncommon but still remain largely unnoticed in geriatric care units. Owing to their relationship with osteoporosis, the majority of fractures occur in elderly females and are frequently bilateral. Besides low back pain, truncated sciatica is mainly observed.

A trans-iliac-sacral-iliac-bar procedure offers an elegant, minimally invasive procedure in presence of osteoporotic bone, which relies on the principles of any fracture treatment: stabilisation and compression.

Although the procedure may require some experience, the results are outstanding vis-a-vis a sudden decrease of the VAS score and analgesics consumption, and an almost immediate improvement of the functional status can be expected.
